# High-Magnification Full-Color Real-Time Stereoscopic Microscopy Based on a Liquid Crystal Polarization Grating

**DOI:** 10.3390/nano16160990

**Published:** 2026-08-11

**Authors:** Jiaoyang Li, Chenhao Li, Zihao Tan, Zhuoming Li, Fujuan Wang, Xiaolan Liu, Xuguang Huang, Jiahui Wang

**Affiliations:** 1 Guangdong Provincial Key Laboratory of Nanophotonic Functional Materials and Devices, School of Optoelectronic Science and Engineering, South China Normal University, Guangzhou 510006, China; lijy27@mail.sysu.edu.cn; 2 National Demonstration Center for Experimental Physics Education, School of Physics, Sun Yat-sen University, Guangzhou 510275, China; lichh66@mail2.sysu.edu.cn (C.L.); lizhm39@mail2.sysu.edu.cn (Z.L.); wangfj@mail.sysu.edu.cn (F.W.); 3 Jiangxi KMAX Industrial Co., Ltd., Nanchang 330100, China

**Keywords:** liquid crystal polarization grating, geometric phase, stereoscopic microscopy, polarization multiplexing, full-color microscopy

## Abstract

Three-dimensional (3D) microscopic imaging is indispensable for fundamental scientific research and clinical medical diagnosis. Given that conventional widefield optical microscopy and standard confocal microscopy fail to realize high-magnification, full-color, real-time stereoscopic imaging simultaneously, we herein propose a single-optical-path 3D microscopic framework enabled by liquid crystal polarization gratings (LCPGs). The LCPG integrated at the sample plane performs polarization-dependent beam splitting to generate paired left and right viewing channels. These two disparity-bearing view channels share a unified imaging optical path compatible with commercial upright microscopes, wherein an active liquid crystal cell modulates temporal view switching for sequential camera acquisition. We further construct a white-light microscopic platform supporting integrated reflection and transmission imaging modes. Two customized LCPGs with lattice periods of 72.6 μm and 56.9 μm are fabricated, offering angular view separations of 0.84° and 1.07°, respectively. Both gratings achieve ±1st-order diffraction efficiencies above 97% with polarization crosstalk not exceeding 0.8%. The developed system acquires paired left-right images with valid binocular disparity, which can be reconstructed into intuitive stereoscopic perceptions via a 3D display monitor. This LCPG-based optical architecture upgrades standard upright microscopes to compact dual-view stereoscopic imaging systems, while fully inheriting the native merits of white-light illumination and high-magnification microscopic observation.

## 1. Introduction

Acquiring three-dimensional (3D) microscale structural information is essential for materials science, biomedicine, micro/nanofabrication, and other interdisciplinary fields. Current microscopic 3D imaging methodologies are broadly categorized into direct stereoscopic imaging and scanning-assisted tomographic imaging. Direct stereoscopic imaging is dominated by conventional stereomicroscopes, which fall into two mainstream configurations: common main objective (CMO) and Greenough geometry. For CMO stereomicroscopes, dual viewing channels share a single large-aperture main objective, while Greenough-type stereomicroscopes adopt two decoupled optical paths to capture samples from disparate viewing orientations. Both configurations rely on a fixed baseline distance to produce valid binocular disparity. Nevertheless, such devices suffer from limited magnification (typically 6–8×), which hinders their implementation in high-resolution microscale characterization. By comparison, conventional high-magnification microscopes adopt high-numerical-aperture (high-NA) objectives with compact optical layouts, where incident and collected light propagate predominantly along a coaxial optical path. This coaxial design yields identical viewing perspectives for paired eyepieces, prohibiting the generation of binocular disparity and stereoscopic perception.

Light-field microscopy offers an alternative 3D imaging strategy, which integrates a microlens array at the image plane to simultaneously record spatial and angular optical information within a single snapshot, facilitating post-capture digital refocusing and volumetric 3D reconstruction [1,2,3]. However, spatial and angular sampling mutually occupy detector pixel resources in this technique, such that elevating angular sampling density will inevitably compromise spatial resolution.

Scanning-based 3D microscopy, represented by optical sectioning techniques such as confocal microscopy [4,5,6,7,8] and scanning-probe techniques such as atomic force microscopy (AFM) [9,10], can provide high-precision depth-resolved or surface topographic information. However, its point-by-point or layer-by-layer scanning process results in relatively low imaging speed, which restricts its application to dynamic samples. In addition, the reconstructed images are often grayscale or pseudo-colored and usually lack full-color stereoscopic visualization.

Recently, planar optical elements have unlocked promising avenues for compact 3D imaging and high-precision depth sensing. For instance, Fan et al. realized visible-band integral imaging via a broadband achromatic metalens array [11], while Guo et al. reported a jumping-spider-eye-inspired single-shot metalens depth sensor for efficient depth perception [12]. Targeting stereoscopic microscopic scenarios, Long et al. incorporated a birefringent metalens into a commercial Greenough-type stereomicroscope to yield high-NA parallax images for left and right viewing channels [13]. Nevertheless, the intrinsic chromatic dispersion of metalenses restricts most metalens-based imaging systems to monochromatic operation or customized chromatic-corrected imaging scenarios [14].

Liquid crystal polarization gratings (LCPGs) are typical geometric-phase optical elements characterized by a continuous and periodic variation in their in-plane optical-axis orientation. Under the half-wave retardation condition, left- and right-handed circularly polarized components are efficiently diffracted into opposite first orders, enabling compact polarization-selective beam splitting. Their high diffraction efficiency, thin and lightweight configuration, and flexible designability make LCPGs promising for beam steering, polarization control, and compact imaging systems [15,16,17,18,19]. However, their use in constructing dual viewing channels for high-magnification, full-color stereoscopic microscopy remains insufficiently explored.

Motivated by these characteristics, we develop a single-path white-light microscopic system enabled by LCPGs, which supports compatible transmission and reflection imaging modes. By introducing polarization-dependent beam splitting into a single-objective microscopic architecture, the proposed system generates paired parallax stereoscopic images. An electrically tunable liquid-crystal cell combined with a polarizer enables temporal selection of dual-view images, and the acquired image pairs can be rendered on a 3D monitor for real stereoscopic observation. Moreover, a deep-learning-based depth estimation algorithm is adopted to reconstruct relative depth maps, enabling qualitative topographical visualization of tested specimens.

## 2. Experimental System

### 2.1. Stereoscopic Imaging Principle

**Figure 1:**
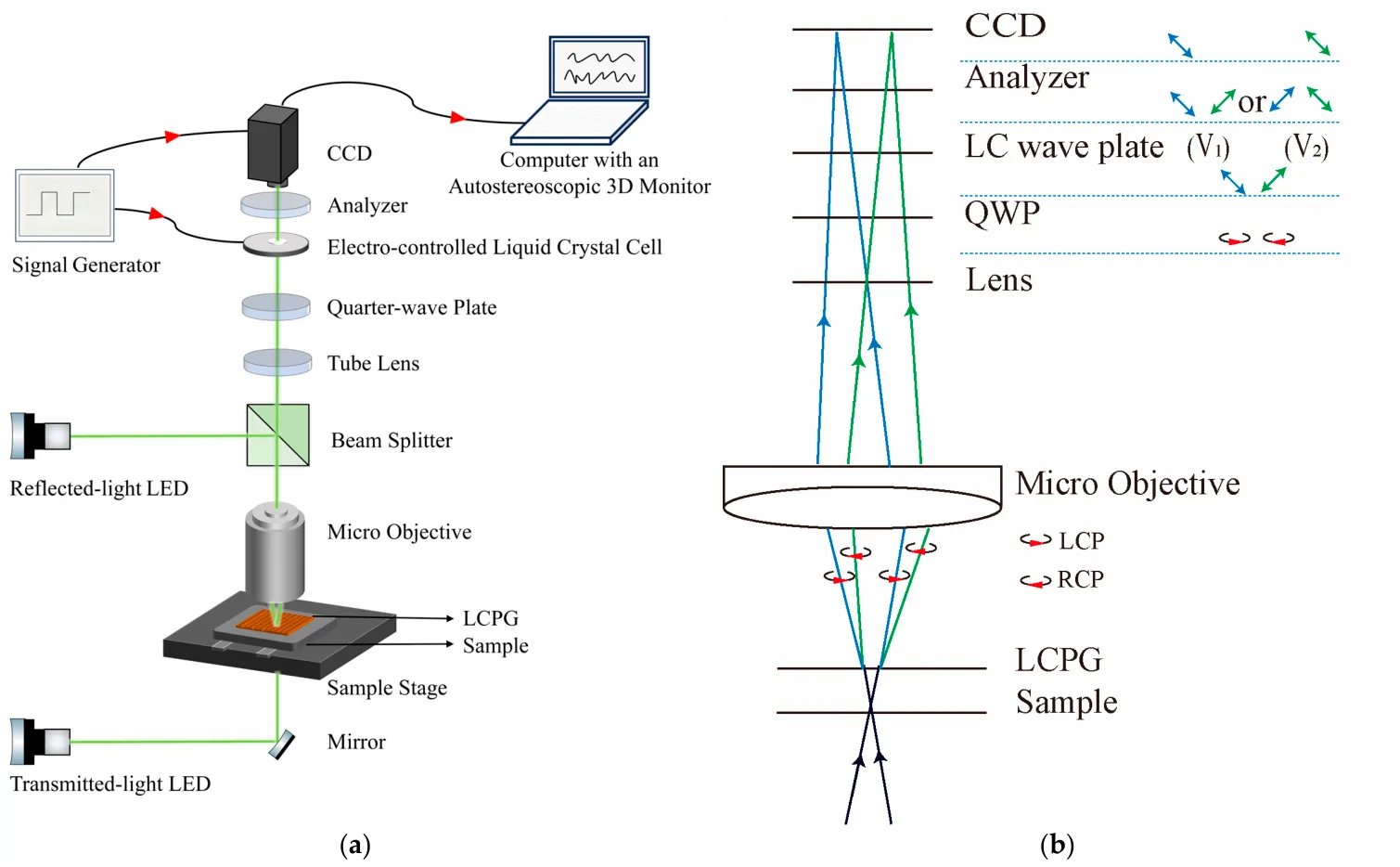
(**a**) Schematic diagram of the optical path in the stereoscopic microscopy system. (**b**) Schematic diagram of the transmitted-light optical path. In (**b**), the blue and green arrows on the left indicate the directions of light propagation, whereas those in the upper-right part indicate the polarization directions of the linearly polarized light.

Stereoscopic imaging reconstructs 3D structural information from multi-view images by leveraging inherent binocular disparity [20,21,22,23]. Conventional stereomicroscopes generate perspective differences via spatially isolated optical axes with a fixed physical baseline. Herein, we realize stereoscopic acquisition on a single-objective microscope via polarization-governed angular beam splitting. As schematically depicted in Figure 1, an LCPG is mounted adjacent to the sample plane, which diffracts left- and right-handed circularly polarized light components toward distinct propagation directions. This configuration constructs a pair of angularly separated viewing channels within one unified optical path, and further generates effective horizontal binocular disparity. The acquired disparity correlates closely with depth-variant sample morphology, which supports valid stereoscopic perception. In addition, enlarging the angular offset between dual viewing channels can amplify binocular disparity and optimize the final stereoscopic viewing effect.

### 2.2. Experimental Setup of the Stereoscopic Microscopy System

A white-light LCPG-based microscopy system capable of both reflection- and transmission-mode imaging was constructed using an optical rail and a sample stage (IMA-50XY/Z, JCOPTIX, Nanjing, China), a pre-collimated white-light LED source (LEM5700C1, JCOPTIX, Nanjing, China), a 20× long-working-distance objective lens (NA = 0.40), a color CMOS camera (MER2-630-60U3C, Daheng Imaging, Beijing, China), and an autostereoscopic 3D monitor (MiD-24VT, Midstereo, Guangzhou, China), as shown in Figure 1a.

The sample under observation was mounted on the sample stage, with the LCPG positioned above it. In reflection mode, the pre-collimated white-light beam was focused onto the sample by the objective lens, and the reflected light was collected by the same objective before propagating through the subsequent optical path. In transmission mode, the sample was illuminated by the white-light beam, and the transmitted light was first split by the LCPG, then collected by the objective lens and recorded by the camera. A 532 nm quarter-wave plate, a voltage-driven liquid-crystal cell, and an analyzer were sequentially placed in front of the camera to enable polarization-selective imaging.

Figure 1b illustrates the principle of polarization-dependent beam-splitting imaging in the transmission-mode system. The liquid-crystal cell was driven by a 1 kHz square-wave carrier, while the driving voltage alternated between two amplitude levels (V_1_ and V_2_) at 5 Hz to switch the liquid-crystal molecules between two orientation states. Consequently, the polarization state of the transmitted light periodically alternated between two orthogonal components. After polarization selection by the analyzer, the left- and right-view parallax images were sequentially recorded by a camera operated in external-trigger mode, with a maximum frame rate of 60 fps. The 5 Hz square-wave modulation corresponded to a theoretical stereoscopic-pair acquisition rate of 5 pairs/s. The time-multiplexed image pairs were then imported into the viewpoint-synthesis software(developed in-house) for the autostereoscopic 3D display. To match the 120 Hz refresh rate of the display, each acquired left- and right-view frame was repeated 12 times, thereby enabling stereoscopic 3D visualization [24].

### 2.3. Design of the LCPGs

Specifically, the adopted LCPG belongs to a typical geometric-phase grating fabricated via polarization-holographic exposure [15,16,25]. A sulfonic azo dye SD1 (Ningcui Optics, Nanjing, China) photoalignment layer is coated on a glass substrate to record the spatially periodic polarization interference field generated by two oppositely handed circularly polarized interfering beams [26,27,28,29,30]. Upon polarized light illumination, SD1 molecules perform photoinduced molecular reorientation, constructing a periodic in-plane alignment pattern perpendicular to the local polarization orientation of the incident exposure field. Benefiting from surface anchoring effects, liquid crystal molecules align conformably with the patterned orientation of the SD1 photoalignment layer. Such interaction enables the continuous and periodic rotation of the in-plane optical-axis azimuth of liquid crystal molecules along the grating period direction, which ultimately forms the functional LCPG. Given a recording wavelength of *λ*, and with *α* denoting the angle between each holographic exposure beam and the normal to the sample plane, the total intersection angle between the two interfering beams is 2*α*, and the spatial period Λ of the resulting polarization interference field is given by:

(1) $$\Lambda =\frac{\lambda }{2\sin\alpha }$$

Under normal incidence, left-handed circularly polarized (LCP) incident light is diffracted into the +1st diffraction order and converted into right-handed circularly polarized (RCP) light. Inversely, RCP incident light is diffracted into the −1st diffraction order with polarization converted to the LCP state. The diffraction efficiencies of the ±1st orders, denoted as $\eta_{\left(\pm 1\right)}$ can be defined as:

(2) $$\eta_{\left(\pm 1\right)}=\sin^{2}(\delta /2)$$

where δ refers to the phase retardation induced by the liquid-crystal layer. Ideally, when δ=π, the +1st and −1st order diffraction efficiencies can approach 100% for matching circularly polarized incident light.

Benefiting from the geometric phase modulated by the liquid-crystal layer, the +1st and −1st-order diffracted beams undergo directional deflection with specific diffraction angles *θ*_±1_. Such angular deflection can be quantitatively solved via the classical grating equation, as formulated in Equation (3):

(3) $$\sin\theta_{+1}=\frac{\lambda }{\Lambda },\sin\theta_{-1}=-\frac{\lambda }{\Lambda }$$

Here, *θ*_+1_ and *θ*_−1_ denote the diffraction angles of the +1st- and −1st-order diffracted beams, respectively, measured relative to the zeroth-order direction.

As indicated by Equations (1) and (3), increasing the intersection angle of two exposure beams decreases the grating period Λ. Therefore, the grating period Λ can be precisely tailored via tuning the beam intersection angle during polarization-holographic exposure. Given that the diffraction angle is intrinsically governed by the grating period, the viewing angular separation of the LCPG can be purposefully designed for targeted stereoscopic imaging demands.

### 2.4. Fabrication of Liquid-Crystal Polymer Polarization Gratings

**Figure 2:**
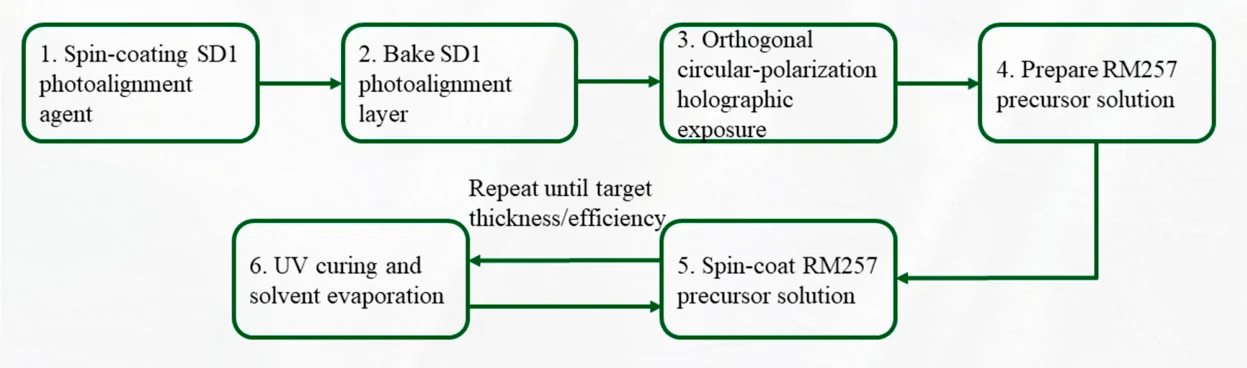
Fabrication flowchart of the liquid-crystal polymer grating.

The LCPGs were fabricated using polarization holography, as illustrated in the fabrication process shown in Figure 2. First, a photoalignment layer of SD1 was spin-coated onto a glass substrate and then baked to remove residual solvent. The SD1 layer was then exposed to the interference pattern generated by two coherent, equal-intensity circularly polarized beams with opposite handedness, inducing a periodic in-plane rotation of the SD1 molecular orientation. This alignment pattern was subsequently transferred to the liquid-crystal molecules through surface anchoring.

The liquid-crystal layer was prepared using RM257 (XINYAO, Nanjing, China) as the reactive mesogen [31,32], propylene glycol monomethyl ether acetate (PGMEA) as the solvent, and IRGACURE 819 (HEOWNS, Tianjin, China) as the UV photoinitiator. The concentration of RM257 in the precursor solution was 9.7 wt.%. The mixed solution was dispensed onto the exposed glass substrate and spin-coated at 2000 rpm for 30 s to form a liquid-crystal layer with the desired thickness. After spin-coating, each RM257 layer was photopolymerized under 365 nm UV irradiation at an intensity of 53 mW/cm^2^ for 100 s, thereby forming a mechanically stable polymerized layer. The UV intensity was measured at the sample plane. The spin-coating and UV-curing procedures were repeated until the designed film thickness was achieved.

**Figure 3:**
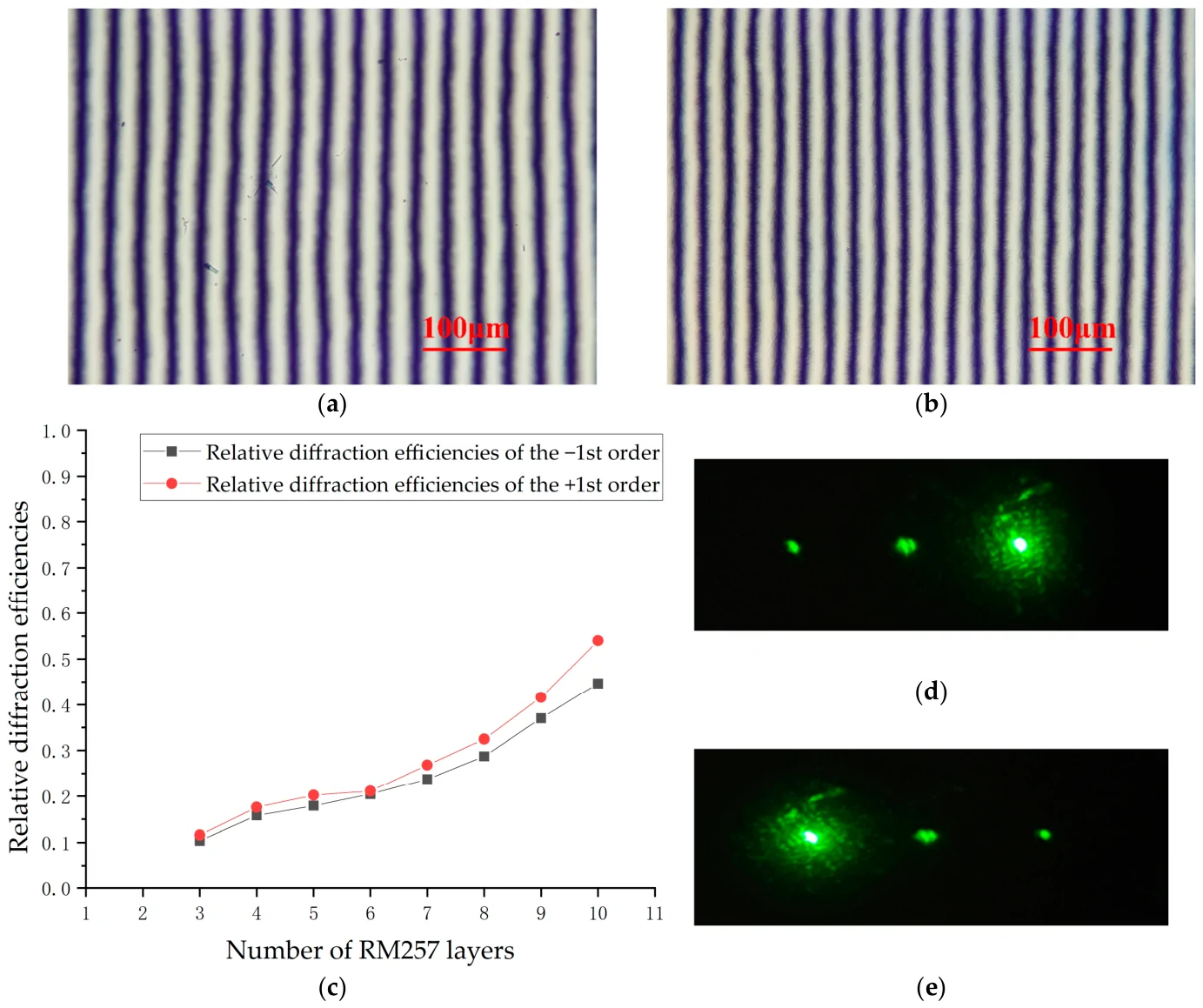
(**a**) Polarizing optical microscopy (POM) image of the LCPG with a period of 72.6 μm. (20× objective; scale bar: 100 μm). (**b**) POM image of the LCPG with a period of 56.9 μm. (**c**) Relative diffraction efficiencies of the +1st and −1st orders. (**d**) LCP Diffraction Pattern. (**e**) RCP Diffraction Pattern.

To achieve the film thickness required for the half-wave retardation condition, multiple spin-coating and UV-curing cycles were performed. During this multilayer fabrication process, the alignment pattern of the previously polymerized liquid-crystal layer was transferred to the newly deposited layer through surface anchoring, enabling the subsequently coated liquid-crystal molecules to preserve the polarization-grating orientation. After each curing cycle, the diffraction efficiency of the polarization grating was measured using a 532 nm laser, and the coating and curing procedures were repeated until the diffraction efficiency exceeded 95%. Finally, two LCPGs with periods of 56.9 μm and 72.6 μm were fabricated. Figure 3a,b shows the polarized optical microscopy images of the fabricated LCPGs observed under crossed polarizers.

The illumination source of the microscopy system is a white-light LED whose emission spectrum is centered in the green spectral region. Therefore, a 532 nm laser was used to characterize the diffraction performance of the fabricated LCPGs. During fabrication, after each RM257 layer was spin-coated and UV-cured, a linearly polarized beam was normally incident on the grating. The optical powers in the +1st, −1st, and 0th diffraction orders were measured, and the relative diffraction efficiencies of the +1st and −1st orders were calculated and plotted as functions of the number of RM257 layers, as shown in Figure 3c. The relative diffraction efficiencies of the ±1st orders are defined as follows:

(4) $$\eta_{\pm 1}=\frac{I_{\pm 1}}{I_{-1}+I_{0}+I_{+1}}\times 100\%$$

As the total film thickness increased, the first-order diffraction efficiencies initially increased and reached their maxima near the half-wave retardation condition. The measured dependence was qualitatively consistent with the theoretical trend, and the 10-layer film, which exhibited the highest total first-order diffraction efficiency, was therefore selected for subsequent experiments.

To measure the relative diffraction efficiencies and polarization crosstalk ratio under LCP and RCP incidence, a quarter-wave plate was used to convert linearly polarized light into circularly polarized light. Under LCP and RCP illumination, most of the incident power was diffracted into the +1st and −1st diffraction orders, respectively, as shown in Figure 3d and Figure 3e. Based on the measured optical powers and Equation (4), the relative diffraction efficiencies of the corresponding +1st and −1st orders were calculated to be 97.5% and 97.6%, respectively. In addition, the angular separations between the diffraction channels associated with the LCP and RCP components were measured to be approximately 0.84° and 1.07°, respectively. The polarization crosstalk ratio of the LCPG is defined as the ratio of the optical power coupled into the undesired first-order diffraction channel to that coupled into the desired first-order diffraction channel under a given circularly polarized incident state, as expressed in Equation (5):

(5) $$C_{\mathrm{LCP}}=\frac{I_{-1}^{\mathrm{LCP}}}{I_{+1}^{\mathrm{LCP}}}\times 100\%,C_{\mathrm{RCP}}=\frac{I_{+1}^{\mathrm{RCP}}}{I_{-1}^{\mathrm{RCP}}}\times 100\%$$

Under normal incidence of LCP and RCP light, the polarization crosstalk ratios of the LCPG were measured to be $C_{\mathrm{LCP}}\;$= 0.8%,${\;C}_{\mathrm{RCP}}=0.7\%,\;$respectively. Since the collimated white-light illumination is generally unpolarized, it can be equivalently treated as a superposition of equal-intensity, mutually incoherent LCP and RCP components. Therefore, a low polarization crosstalk ratio is crucial for the effective separation and independent imaging of the two polarization-dependent viewing channels.

## 3. Results and Discussion

### 3.1. Spatial Resolution Characterization of the Stereoscopic Microscopy System

**Figure 4:**
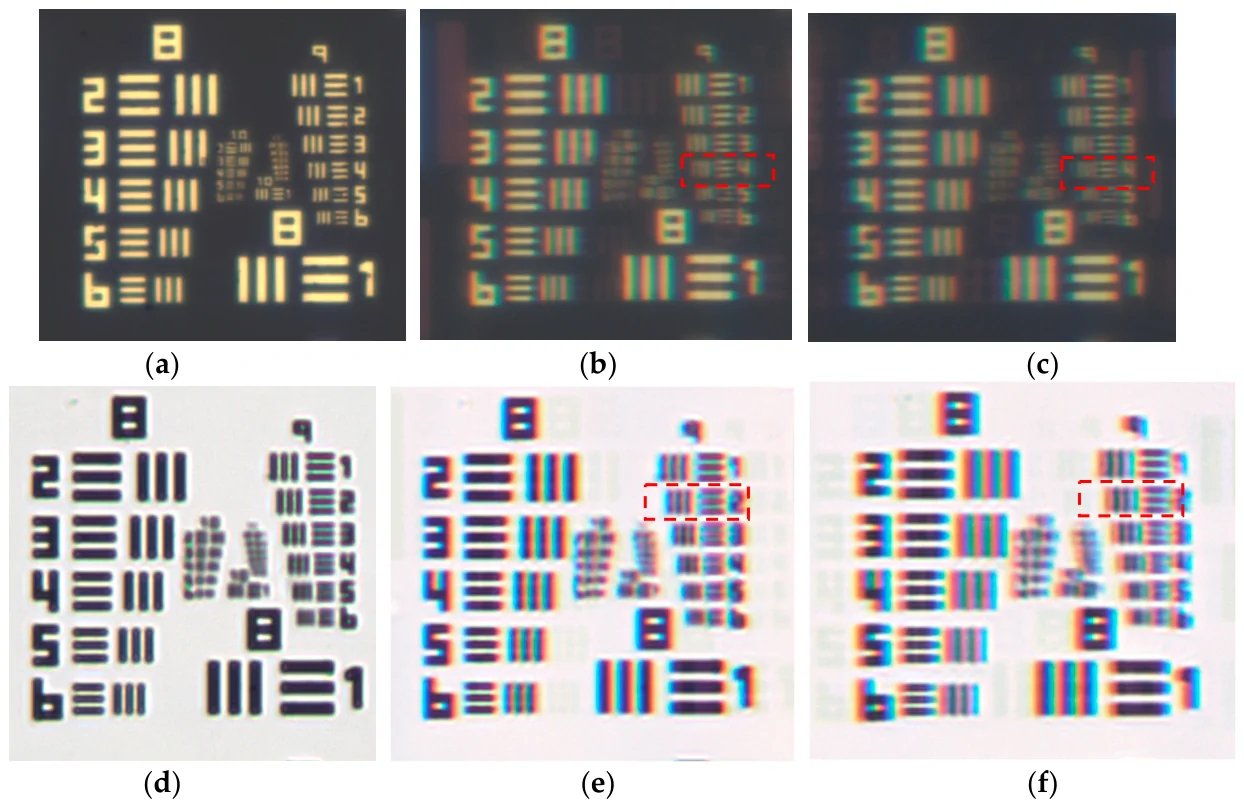
Images of the USAF resolution test chart with the microscope system. (**a**) Reflection mode without LCPG. (**b**) Left-view in Reflection Mode. (**c**) Right-view in Reflection Mode. (**d**) Transmission Mode without LCPG. (**e**) Left-view in Transmission Mode. (**f**) Right-view in Transmission Mode. The numbers indicate the group and element numbers of the HIGHRES-2 resolution target, and the horizontal and vertical line patterns in each element are used to evaluate the spatial resolution of the imaging system. The line width of Group 8, Element 1 is 1.953 μm in each image. The dashed red frames indicate the groups that are just resolvable in the image.

The system employs a 20× objective lens with an NA of 0.40. According to the Rayleigh criterion, the theoretical lateral resolution is given by d = 0.61λ/NA. At a central wavelength of λ = 550 nm, the theoretical lateral resolution of the objective lens is approximately 0.84 μm. A high-resolution target (HIGHRES-2, Newport, Irvine, CA, USA) was used to evaluate the imaging performance of the microscopy system. As indicated by the red boxes in Figure 4, the finest resolvable elements are Group 9, Element 4 in reflection mode and Group 9, Element 2 in transmission mode. These elements correspond to spatial frequencies of approximately 724 lp/mm and 575 lp/mm and line widths of approximately 0.691 μm and 0.870 μm, respectively. These resolution estimates are further supported by the intensity profiles extracted perpendicular to the bar patterns. In white-light imaging, shorter-wavelength components contribute to the detection of higher spatial frequencies. In addition, the chromium patterns of the resolution target provide higher contrast in reflection mode, enhancing the visibility of fine features. The measured resolutions are close to those obtained from the system without the LCPG, indicating that the introduction of the LCPG has a negligible impact on imaging performance.

### 3.2. Imaging Results and Disparity Analysis

**Figure 5:**
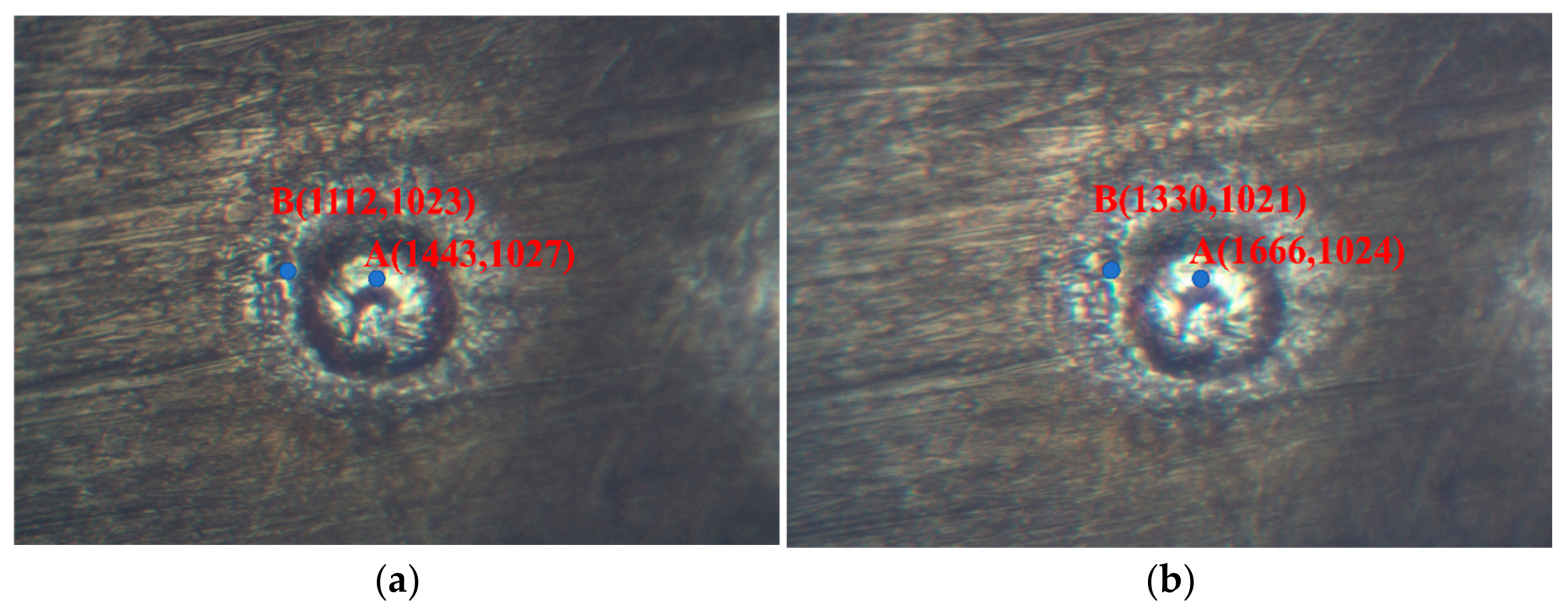

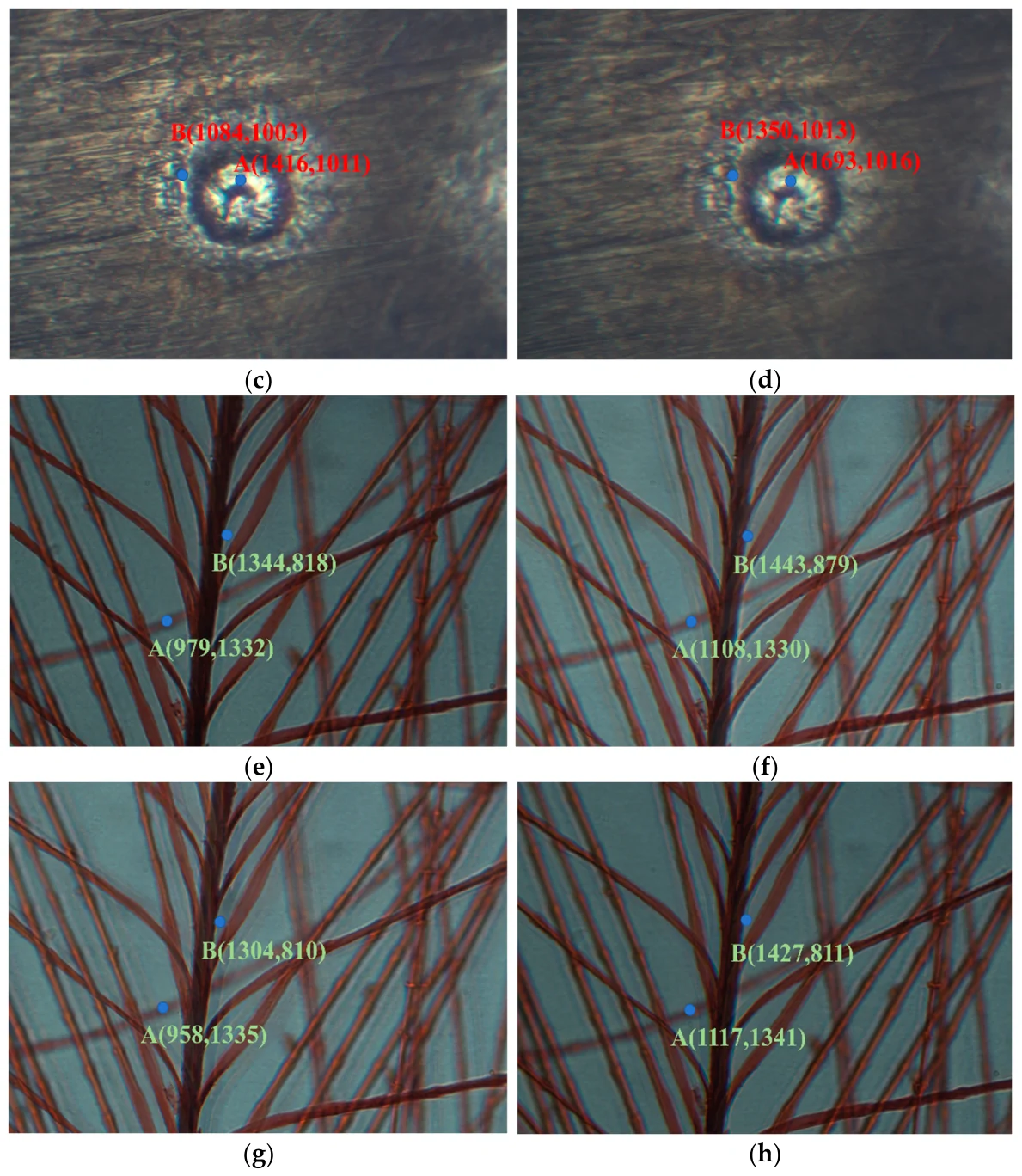
(**a**) Left- and (**b**) right-view images of laser ablation pits with small parallax, captured in reflection mode. (**c**) Left- and (**d**) right-view images of laser ablation pits with large parallax, captured in reflection mode. (**e**) Left- and (**f**) right-view images of paramecia with small parallax, captured in transmission mode. (**g**) Left- and (**h**) right-view images of paramecia with large parallax, captured in transmission mode. The capital letters A and B and the blue dots indicate two selected points at different depths in these images. Point A represents the deeper point, whereas point B represents the shallower point.

Picosecond laser ablation pits on an aluminum plate were selected as reflective samples, while a mounted feather specimen and a stained Paramecium slide were used as transmissive samples. Left- and right-view image pairs of these specimens were acquired using the proposed stereoscopic microscopy system, as shown in Figure 5. To compare the disparities generated by the two LCPGs with larger and smaller periods, denoted as PG1 and PG2, respectively, two representative feature points located at different axial depths were identified in the left and right images. The disparity difference between the two depth positions was then calculated from the horizontal pixel-coordinate differences of the corresponding feature points. The results are summarized in Table 1.

As shown in Figure 5 and Table 1, the proposed system successfully captures dual-view images with clearly observable disparity in both reflection and transmission modes. Under the adopted left–right view arrangement and image-coordinate convention, the system produces positive disparity, causing the reconstructed object to appear behind the plane of the 3D display. Specifically, the measured horizontal disparity is larger for features located at greater depths than for those at shallower positions. Moreover, the large-disparity configuration produces a greater disparity difference between the two depth positions than the small-disparity configuration. This observation is consistent with the principle of stereoscopic imaging: increasing the parallax angle, or equivalently the effective baseline, enhances the disparity variation associated with a given depth difference.

**Figure 6:**
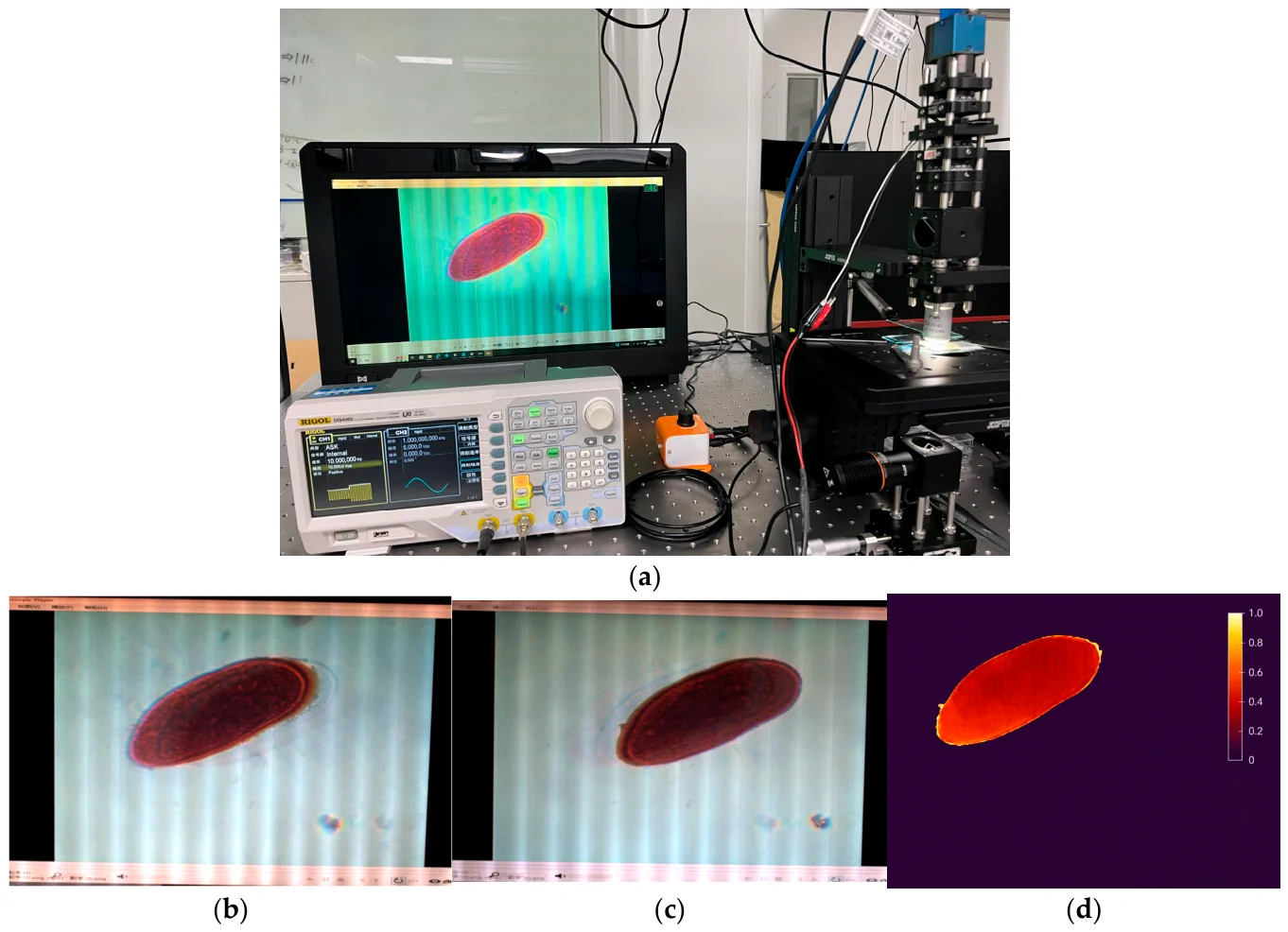
(**a**) Experimental setup for the stereoscopic microscopy system. The captured left- and right-view parallax images are displayed on an autostereoscopic screen, allowing users to perceive full-color, high-definition stereoscopic microscopic images without wearing any auxiliary viewing devices. (**b**) Left-view parallax image. (**c**) Right-view parallax image. (**d**) Real-time relative depth map generation of the sample using a deep learning algorithm.

The left- and right-view image pairs were imported into the viewpoint-synthesis software of the 3D display, producing a visual effect with clearly perceivable stereoscopic depth, as shown in Figure 6a–c.

Deep-learning-based stereo matching has been extensively investigated for stereo depth estimation [33]. In this work, we employed the publicly available IGEV-Stereo algorithm proposed by Xu et al. [34] to estimate the disparity between the acquired left- and right-view microscopic images and generate relative depth maps of the samples in real time, as shown in Figure 6d. The left- and right-view image pair was input into the IGEV stereo-matching network. The network estimated the pixel-wise horizontal disparity by constructing and iteratively updating a geometry-encoding volume. The resulting disparity map was normalized and converted into a pseudocolor image to visualize the relative depth distribution. Because the two parallax views are generated and sequentially acquired within a single-objective, single-camera architecture, conventional intrinsic and extrinsic calibration procedures developed for binocular camera systems cannot be directly applied. Consequently, the disparity-to-depth relationship has not yet been established, and the present results represent relative depth rather than absolute depth in micrometers. In Figure 6d, the color bar represents the normalized relative depth on a scale from 0 to 1, with larger values indicating regions farther from the imaging system, as evidenced by Table 1.

### 3.3. Performance-Determining Factors and Limitations

The performance of the proposed stereoscopic microscopy system is governed by several optical and temporal factors. First, the angular separation between the two viewing channels Δθ is determined primarily by the LCPG’s grating period Λ and illumination wavelength λ, approximately following Δθ ≈ 2λ/Λ. Decreasing the grating period increases the angular separation and stereoscopic disparity; however, the usable angular separation is limited by the objective’s numerical aperture and clear aperture. Moreover, the parallax angle of the present system is not continuously tunable. Instead, discrete parallax angles can be selected by replacing or switching between LCPGs with different grating periods, analogous to switching objective lenses with different magnifications in a conventional microscope. Second, because the diffraction angle and phase retardation of the LCPG are wavelength-dependent, the system exhibits chromatic angular dispersion and wavelength-dependent diffraction efficiency under white-light illumination. Nevertheless, within the wavelength range and experimental conditions used in this study, the resulting dispersion remained within an acceptable range. Third, because the two views are acquired sequentially, rapid target motion between the two acquisition states may cause temporal mismatch and disparity artifacts. The effective stereoscopic update rate is determined by the response time of the liquid-crystal cell, the switching and synchronization scheme, the camera exposure and frame rate, and the computation time of the disparity algorithm.

## 4. Conclusions

In this work, we propose a high-magnification, full-color, real-time stereoscopic microscopy approach based on LCPGs. By introducing an LCPG at the sample plane, polarization-dependent beam splitting is achieved, generating left- and right-view perspectives through diffraction. The disparity image pair carried by the LCP and RCP diffraction beams is combined into a single optical path, analogous to that of a conventional upright microscope. An active liquid-crystal cell is then used to switch between the two polarization states, enabling the left- and right-view images to be sequentially recorded by the camera. Based on this principle, we constructed a white-light stereoscopic microscopy system capable of both reflection- and transmission-mode imaging.

Experimental results show that the two fabricated LCPGs have periods of 72.6 μm and 56.9 μm, corresponding to the smaller- and larger-disparity configurations, respectively. The diffraction efficiencies of the desired first-order diffraction channels exceed 97%, while the polarization crosstalk ratios remain below 0.8%. The two LCPGs generate angular separations of 0.84° and 1.07° between the diffraction channels associated with the left- and right-handed circularly polarized components, respectively. These angular separations provide two distinct viewing angles for recording the left- and right-view images of the target. The system achieved a theoretical stereoscopic-pair acquisition rate of five pairs/s, and each acquired image pair was repeated 12 times for presentation on a 120 Hz autostereoscopic 3D monitor. The developed system achieves a lateral resolution of 0.691 μm in reflection mode and 0.870 μm in transmission mode, while providing clearly perceivable stereoscopic microscopic visualization. In addition, relative depth maps of the samples can be generated in real time using a deep-learning algorithm, further enhancing the visualization and interpretation of stereoscopic structural information.

Compared with conventional approaches, the proposed method enables stereoscopic microscopic imaging within a single optical path while maintaining high spatial resolution, thereby avoiding the structural complexity and alignment challenges associated with multi-path configurations. The LCPG-based architecture further extends conventional upright microscopy to compact dual-view stereoscopic visualization, providing an effective route toward high-resolution, full-color, real-time stereoscopic microscopy. Its compatibility with both reflection and transmission modes, together with real-time depth visualization enabled by deep learning, highlights its potential for a wide range of applications in biomedical research, education, and industrial inspection.

## Figures and Tables

**Table 1:** Disparity analysis for large- and small-period grating-based stereoscopic microscopy (disparity unit: pixels).

Mode	Disparity of PG1		Relative Disparity Variation of PG1	Disparity of PG2		Relative Disparity Variation of PG2
	Deep Point A	Shallow Point B		Deep Point A	Shallow Point B	
Reflection	223	218	5	277	266	11
Transmission	129	99	30	159	123	36

## Data Availability

Data are contained within the article.
